# How do geriatricians practise comprehensive geriatric assessment in the outpatient setting: an analysis of geriatricians’ letters and a comparison to the Medicare benefits schedule requirement

**DOI:** 10.1111/imj.16535

**Published:** 2024-10-10

**Authors:** Sarah T. Fox, Olivia Demichelis, Constance Dimity Pond, Monika Janda, Ruth E. Hubbard

**Affiliations:** ^1^ Centre for Health Services Research University of Queensland Brisbane Queensland Australia; ^2^ Internal Medicine Services The Prince Charles Hospital Brisbane Queensland Australia; ^3^ Wicking Dementia Research and Training Centre University of Tasmania Hobart Tasmania Australia; ^4^ Geriatric Medicine University of Queensland Brisbane Queensland Australia; ^5^ Geriatric Medicine Princess Alexandra Hospital Brisbane Queensland Australia

**Keywords:** geriatric assessment, outpatient, frailty, geriatrician, quality of life

## Abstract

**Background:**

Little is known about what components geriatricians routinely incorporate into outpatient comprehensive geriatric assessments (CGAs).

**Aims:**

This study explored what components of CGAs are routinely incorporated into geriatricians’ letters and assessed their consistency with the Medicare Benefits Schedule (MBS) and a recently published survey of geriatricians.

**Methods:**

We completed a manual content analysis, supplemented by qualitative thematic analysis, of 34 letters from five geriatricians, collected as part of the GOAL Trial.

**Results:**

While more than 80% of letters included each of the key clinical domains described in the Medicare Benefits Schedule and survey of geriatricians, only 62% included advanced care planning and 47% mentioned immunisations. Forty‐seven percent of letters included goal setting. Few letters showed evidence of multidisciplinary working. Issues identified by the geriatrician centred around the themes of advance care planning, symptom identification and management, medical comorbidities, strategies to support quality of life and interventions to manage frailty. Patient concerns identified in the letters were cognition and mood, declining function, future planning and symptom management.

**Conclusions:**

Analysis of geriatricians’ letters provides important and novel insights into usual CGA practice. The letters provide evidence of multidimensional assessments of physical, functional, social and psychological health, and most include use of standardised tools. However, less than 50% include evidence of goal setting or multidisciplinary working. The results allow consideration of how CGAs might be carried out in the outpatient setting, so that interventions focused on improving the quality and efficacy of this intervention can be implemented.

## Introduction

Comprehensive geriatric assessment (CGA) is an evidence‐based model of healthcare for older adults, particularly for those living with frailty, multimorbidity, functional impairment or psychosocial vulnerability.^1^ CGA involves identifying medical, social and functional needs of patients and developing a co‐ordinated care plan to meet those needs using a multidisciplinary approach.^2^ CGA primarily focuses on functional improvement, care planning and personalised care. It is a complex healthcare intervention that requires adaptation to different settings, resource availabilities and patient requirements.^3^ CGA delivered in the inpatient setting has been associated with reduced mortality and increased chance of returning to independent living after hospital discharge.^4^ However, the evidence for CGA delivered in the outpatient setting is less robust.^5^ It is unclear what drives these differences in efficacy, although it is likely that CGA delivered in the outpatient setting, has fundamental differences to CGA delivered in the inpatient setting. For example, the core processes of multidisciplinary working and care coordination likely look different in the inpatient versus outpatient space. However, no research has been able to interrogate these differences in a meaningful way.

Prior literature attempted to describe minimum criteria for the intervention while acknowledging the necessary prerogative for CGA to adapt to different patients and contexts. For instance, the Australian government Medicare Benefits Schedule (MBS) stipulates that to be eligible for financial reimbursement,^6^ CGA must be a multidimensional assessment encompassing the medical, psychological and social aspects of health. Required domains include psychological, socioeconomic, medical history, immunisations, medicines, function, cognition, mood and advance care planning. According to the MBS criteria, standardised tools should be used as appropriate, effort should be made to understand the perspective of the carer or family, the clinician must formulate an issues list and management plan, short‐ and long‐term goal setting should occur, and the clinician should communicate to the patient the assessment and management plan.^6^

To understand the minimum criteria for CGA (including both inpatient and outpatient CGA) from the perspective of clinicians, we previously surveyed geriatricians in Australia and New Zealand (the survey).^7^ While most geriatricians agreed with the MBS criteria domains, fewer than 80% of respondents thought advance care planning should be included in all or most CGAs, 55% of respondents felt goal setting was a core feature of CGA, and 60% felt that standardised tools were a core feature.^7^ Less than 60% of surveyed geriatricians felt that ‘multidisciplinary involvement’ was a core feature of CGA, in direct contrast to a broadly accepted definition of CGA.^2^, ^7^ Therefore, a discrepancy currently exists between geriatricians’ opinions about CGA and government funding criteria. It is unknown how that contributes to clinical practice.

Older adults with chronic kidney disease (CKD) experience a high prevalence of frailty and other comorbidities, which impacts their quality of life.^8^ As such, it has been suggested they may benefit from CGA, but the evidence for CGA in the outpatient setting has not been established. Little is known about how geriatricians routinely practise CGA in the outpatient setting.

### Objective

The Australian Government has set out minimum criteria for CGA (‘the MBS’), and evidence exists for geriatricians’ opinions about how CGA works best in practice (‘the survey’).^6^, ^7^ However, to what degree CGA is practised in accordance with these minimum funding criteria and geriatrician opinions remains unclear. By studying geriatrician letters to general practitioners (GPs), collected as part of a cluster randomised controlled trial of CGA for frail outpatients with CKD (the GOAL trial), we aim to understand which components of CGA are routinely incorporated into geriatricians’ letters and to compare this with the MBS and the survey.^6^, ^7^, ^9^ In this study, the letters are used as a proxy, in the absence of being able to directly observe geriatrician consultations. We also aim to undertake an exploratory analysis of how geriatricians describe the clinical issues, patient perspectives and patient goals in their letters.

## Methods

### Ethics

This study received ethics approval from Metro South Human Research Ethics Committee (HREC/2020/QMS/62883).

### Sampling

The GOAL Trial was a cluster randomised controlled trial conducted across six states in Australia investigating whether outpatient CGA could increase attainment of personal goals for people living with frailty and CKD.^9^ The results of the trial are not yet available, but the GOAL Trial protocol has been published.^9^ The trial did not stipulate a form or structure for the CGA, rather asking that geriatricians provide CGA as per their usual processes. Each of the seven intervention sites in the GOAL Trial were asked to provide the deidentified geriatrician letters from five CGA consultations, randomly selected by the research coordinator for the trial site. The number and sampling method were chosen for convenience for trial sites. Geriatricians did not have a say as to which patient letters were selected. One site only recruited four patients in total and so was asked to provide the letter for each of these patients. Each letter sampled related to a unique patient and were for the initial CGA encounter only. Letters from follow‐up or review appointments were not analysed.

### Quantitative manual content analysis

A quantitative manual (human‐coded) content analysis was designed to address the first research question.^10^ A codebook, which reflected the key components of the survey and the MBS domains, was developed for data analysis (Appendix I).^6^, ^7^ A total of 51 CGA letter components were included in the codebook. Forty‐five primary items were components drawn directly from the letters as dichotomous ‘component included’ or ‘component not‐included’ variables. Three items were free text responses to record which standardised tests (e.g. Mini‐Mental State Examination (MMSE)) or frailty screening tools (e.g. Clinical Frailty Scale) were used, and one item was the count of standardised tools mentioned in each letter. Three more items were combined items, marked ‘yes’ if multiple components were all included in a letter, such as a letter being marked as ‘structured’ if it included an issues list, management plan and at least three headings. The criterion ‘patient told’ referred to whether the letter made clear what had been communicated to the patient. It was marked as ‘yes’ if the letter mentioned that the impression, issues or management plan was discussed with or communicated to the patient. It was marked as ‘no’ if there was no mention of what was communicated to the patient.

The first author extracted data from all letters using this quantitative manual analysis codebook. The second author then independently coded 20% of the letters against the 45 primary items. Discrepancies in coding were clarified between the two researchers. If differences in item coding occurred, the first author coded that item across all letters. The first author then reviewed these results and made the final coding decision.

To ensure comparability between the survey of geriatricians and the MBS CGA criteria, we created definitions of ‘multidisciplinary’ and ‘structured assessment’. A letter was deemed to have multidisciplinary input if a referral was made to two or more allied health professionals. A CGA was deemed ‘multidimensional’ if the letter included all of medical, functional, psychological and social domains as stipulated in the MBS. When comparing to the survey we could not use this ‘multidimensional’ component because the survey asked whether a core component of CGA was ‘assessment of multiple different domains’, rather than defining the number or type of domains. Therefore, when comparing to the survey, we included multidimensional to mean including medical history, medicines history and at least one other domain.

### Reflexive thematic analysis

A reflexive thematic analysis was designed to explore how geriatrician letters described the identified issues, patient concerns and patient goals. Reflexive thematic analysis was chosen as a method for its flexibility and ability to explore the data with depth in an inductive fashion.^11^ The qualitative analysis was predominantly undertaken by the first author. Reflexive journalling and memoing was undertaken in NVivo 14.^12^

The first author is a Caucasian woman residing in Brisbane Australia. She is a specialist consultant geriatrician and general physician. She is completing a PhD under the supervision of other co‐authors. Her research focus is currently a process evaluation of the GOAL Trial investigating the efficacy of CGA delivered in the outpatient setting, for patients with CKD. SF has no personal lived experience as a patient who receives CGA. She practices CGA in her role of geriatrician. This will have influenced her reading of the letters and assisted her understanding of the content.

The NVivo 14 software was used to support storage and analysis of qualitative data.^12^ An audit trail was produced to show the progression of ideas, from a descriptive through to an interpretive and analytical approach during the analysis cycle. The various phases of the thematic analysis are discussed in more detail in what follows, with reference to the six phases originally described by Braun and Clark, with further reference to their updated guidance.^11^, ^13^ An audit trail of these six phases was maintained in NVivo 14.^12^ The coding frameworks for phases 2–5 are included as Appendices II, III, IV, V.

In phase 1, the researchers familiarised themselves with the data. Redacted geriatrician letters for analysis were downloaded into the NVivo software. In the first phase they were read carefully, utilising reflexive journalling in the form of NVivo memos and annotations to ensure active engagement with the material. These memos and annotations were used to highlight thinking about the issues and emerging concepts and ideas. A mind map was created at this phase to brainstorm and frame initial thinking about the material. In phase 2, the researcher conducted open coding wherein codes were generated from the raw data in an inductive manner. At this point they were not organised hierarchically but drawn descriptively from the data. Subsequently, in phase 3, the researchers searched for themes using an interpretive approach to organise the initial codes into early themes, under the umbrella of geriatrician issues, patient concerns and patient goals. In phase 4 the researchers used the themes identified in phase 3 and began forming them into broader groupings, within the three qualitative research questions. They also deconstructed some of the themes into more refined codes and grouped subthemes that dealt with similar concepts. The lead author then read all the text references within the developed themes to assess for coherence and to ensure they reflected the contents of the original letters. During phase 5 further interpretation and abstraction consolidated the themes into the final thematic framework.

Finally, in phase 6, the report was written in a narrative sense with reference made to the original quotes (Appendix VI).

## Results

### Demographics

Of the 34 patient letters that were coded, 16 (47%) of the patients were female, 17 (50%) were male and one (3%) was unknown.

### Manual content analysis: comparison of letters with MBS criteria

Seventy‐six percent (26/34) of the analysed letters included all four of the key MBS domains (physical, psychological, social and medical), and 76% (26/34) included a standardised, validated tool (such as the MMSE). The carer was identified in 73% (11/15) of patients who were dependent for activities of daily living, but the carer or family concerns were only identified in 6% (2/34) of letters. The results of the manual content analysis as related to the MBS criteria are further presented in Table 1. In 16/34 (47%) letters, patient goals were discussed in general terms. None of the letters broke goals down into short‐ and long‐term goals. In 32/34 (94%) letters a management plan was specified. These management plans included a range of suggestions, including medicine changes, medical investigations, referrals to other health professionals and lifestyle changes.

**Table 1:** Manual content analysis of geriatrician letters as per the domains set out in the Medicare Benefits Schedule (MBS) criteria for comprehensive geriatric assessment (CGA)

MBS CGA criteria	Manual content analysis of 34 letters, *n* (%)
Domains	
Medical history	34 (100)
Function	34(100)
Medicines	31 (91)
Psychology/psychiatry	30 (88)
Cognition	28 (82)
Socioeconomic	25 (74)
Advanced care planning	21 (62)
Immunisations	16 (47)
Issues list	26 (76)
Management plan	32 (94)
Goals	
Goal setting	16 (47)
Long‐term and short‐term goals	0 (0)
Is it clear what the patient's been told?	9 (26)

### Manual content analysis: comparison with survey of geriatricians in Australia and New Zealand

**Figure 1:**
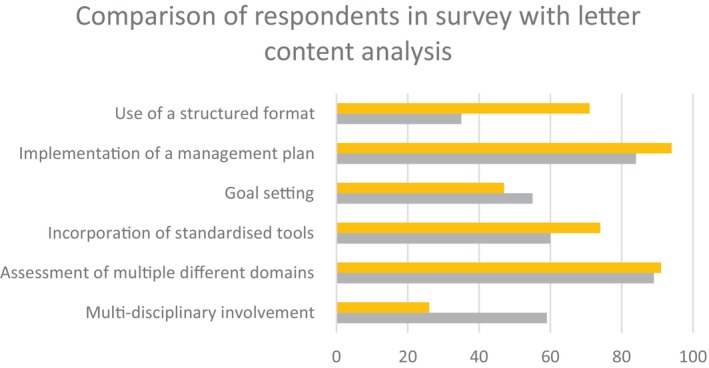
Comparison of results of manual content analysis of geriatrician letters (‘Letter Analysis’), versus opinions of geriatricians surveyed about their views of comprehensive geriatric assessment (CGA) (‘Survey Respondents’),^7^ with respect to core features of CGA. The *x*‐axis is the percentage of letters that met this criterion (yellow bar) and the percentage of survey respondents who named these criteria as core features of CGA (grey bar).

Fifty‐six percent of letters included referral to one or more allied health professionals, whereas 44% did not. Only 26% of letters included referrals to at least two allied health professionals, meeting our ‘multidisciplinary’ criterion to allow comparison with the previous survey results. This contrasts to the survey in which almost 60% of respondents identified multidisciplinary involvement as a core feature of CGA.^7^ Seventy‐one percent (71%) of letters were classified as ‘structured’, meaning they included an issues list, management plan and at least three headings within the letter. This contrasts to the survey in which only 35% of respondents felt that ‘use of a structured format’ was a core feature of CGA.^7^ Ninety‐one percent (91%) of the letters included assessment of multiple domains as defined as medical history, medicines review and at least one other domain. This aligns with the survey in which 89% of respondents felt that ‘assessment of multiple different domains’ was a core feature of CGA^7^ (Fig. 1; Table 2).

**Table 2:** Comparison of results of the manual content analysis of geriatrician letters (‘Letter Analysis’), versus the opinions of geriatricians surveyed about their views of comprehensive geriatric assessment (CGA) (‘Survey Respondents’),^7^ with respect to the domains assessed in CGA

Domain	Survey respondents: percentage of respondents who said these domains should be assessed in ‘all’ or ‘most’ patients undergoing CGA	Letter analysis: manual content analysis of 34 letters, *n* (%)
Medical history	99%	34 (100)
Medicines	100%	33 (97)
Clinical examination	98%	26 (76)
Falls history	96%	33 (97)
Function	99%	34 (100)
Continence	91%	26 (26)
Cognition	98%	28 (82)
Mood	95%	30 (88)
Socioeconomic	100%	25 (74)
Alcohol/substances	96%	24 (71)
Religious beliefs	33%	2 (6)
Advance care planning	78%	21 (62)
Capacity assessment	19%	1 (3)
Collateral history	87%	11 (32)

### Qualitative analysis

The results of the reflexive thematic analysis are summarised in Table 3.^11^ Themes that geriatricians used to describe issues in their letters included advanced care planning, symptom identification and management, identification and management of medical comorbidities, strategies to support independence and quality of life and strategies to manage frailty and functional decline. Geriatricians described patient concerns centred around themes of cognition and mood concerns, declining function and difficulty managing activities and daily living, future planning and symptoms management. Goals were described in groups relating to improvement of health status, increasing engagement and maintenance of independence and personal agency.

**Table 3:** Qualitative analyses of three research questions (RQ)

Themes	Codes	Illustrative quote
RQ1: How do geriatricians describe patient issues in their letters?		
1.1 Advance care planning	Substitute decision‐makers	Please support with enduring power of guardianship
	Planning for future medical care	Today her preference and that of her family has been that she would, for example, be for resuscitation or intubation if she becomes acutely unwell. As she accumulates morbidity and becomes more frail, this might no longer be what is in her best interest or align with her goals and likely benefit, and this will need to be discussed again if she presents acutely unwell.
	Planning for future care requirements	…has not completed any advanced care planning and has not registered with the My Aged Care Program.
1.2 Symptom identification and management	Fatigue and breathlessness	…may benefit from energy/breath conservation techniques, which pulmonary rehabilitation could provide.
	Non‐fatigue symptoms	…she is due for follow‐up with pain specialist, which from a quality of life (QOL) point of view is one of the more important things that can happen for her.
1.3 Identification and management of medical comorbidities	Cognitive impairment diagnosis and strategies to maintain cognitive health	Mild cognitive impairment, likely vascular aetiology.
	Identification and management of non‐cognitive comorbidities	She should also have assessment for osteoporosis especially in light of her renal disease and her report of a fall last year. Please consider BMD [bone mineral density] assessment and treatment for osteoporosis if indicated.
1.4 Strategies to support independence, QOL and psychosocial well‐being	Low mood including psychological adjustment to changed health status	She and her husband are socially isolated and her mood is affected by this isolation and the relative hardship of her life over the past few years.
	Psychosocial strategies to improve quality of life	Encouraged to pursue his interests – previously avid singer and guitar player but gave it up due to loss of dexterity/grip in his fingers
	Strategies to support independence or functioning	An OT home safety assessment would be beneficial for him including the use of sensor lighting.
1.5 Strategies to manage frailty and functional decline	Mobility and falls	She is a significant falls risk, which is likely to be multifactorial due to her poor balance, neuropathy, central aetiology and polypharmacy. She would benefit from a structured exercise program.
	Frailty and functional decline	In terms of frailty and the maintenance of independence, [patient] would strongly benefit from regular exercise and an endurance program. I have advised him to discuss with his dietician protein supplements.
RQ2: How do geriatricians describe patient concerns in their letters?		
2.1 Cognition or mood concerns	Cognition	…says that she is becoming forgetful in the past 2 years and will forget what she has done or would forget appointments if not reminded.
	Mood	feels loneliness … on antidepressant … used to see psychiatrist but too expensive did find it helpful to talk to someone GP has done mental health care plan – has seen psychologist
2.2 Declining function and difficulties with managing activities of daily living	Falls	…has not had any falls and self‐develops strategies to minimise his falls risk. He feels that his lifestyle is quite sedentary, and he is trying to walk more. He does not engage in formal exercises. He mainly wears thongs, and I have advised him on the importance of appropriate footwear.
	Physical slowing and functional decline	…reports that she has become progressively frail since about a year after renal transplant. She said she was ‘wonderful’ for about a year after transplant but then got weaker, and she is frustrated now by her lack of physical energy and increasing dependence.
2.3 Future planning	Barriers to accessing care and support	Financial situation to be improved. Needs a new bed and needs to be able to afford transport to dialysis; living in retirement village is expensive.
	Concern around how he will meet responsibilities in future	… worries that he would like his wife to be supported in the event that he has a deterioration in his health. He feels confident that his family would support his wife, but he is in the process of trying to introduce her to aspects of their shared finances and other duties that he usually takes care of, such as filling cars up with petrol and vehicle maintenance.
	Impact of medical care on QOL	The first issue of concern is regarding his recent commencement of dialysis about 6 months ago on a background of chronic kidney disease for the last 5 years or so. He feels this has taken a bit of a toll on his physical well‐being, which is understandable
2.4 Symptom management	Fatigue and dyspnoea	Reports being very fatigued since December/January last year Significantly reduced ET – now dyspnoeic
	Non‐fatigue symptoms	He describes his sleep as ‘difficult’. He usually goes to bed late, around 2200–2400, and sleeps for about 2–3 h before waking up, mostly with leg cramp pain.
RQ3: How do geriatricians describe patient goals in their letters?		
3.1 Improve health status	Lifestyle modification to improve health or fitness	…trying to go to swim classes twice per week – making it maybe once per week.
	Medical treatment	…is compliant with his therapy and hoping to receive a kidney transplant in the future.
3.2 Increase or maintain engagement	Social engagement	…to maintain her social outings with weekly lunch with family and friends. She loves social group activities.
	Work or leisure activity	…main goals at present are to continue to remain in her own home in the community and for her renal function to remain stable, so that she can continue her hobby creating soft toys and quilts for donation to disadvantaged children overseas.
3.3 Maintain independence and personal agency	Increase support	assistance with house cleaning if possible. Currently her son is helping her with house maintenance.
	Live independently	He would like to remain as independent as possible and still actively participates in his business.

## Discussion

This study used geriatricians’ letters as a proxy for understanding the contents of CGAs in an outpatient setting. The letters reviewed for this study showed evidence of multidimensional assessments with a focus on function, including the effect of cognitive, emotional and physical health on patients' functional ability in activities of daily living and incorporation of standardised tools. Compared to the stipulations of the MBS, the letters less commonly included advance care planning and goal setting and did not make it clear whether the patient had been involved in decision‐making or been informed of the outcome of the assessments. Furthermore, in contrast to a commonly accepted definition of CGA in the literature, the letters were notable for their lack of evidence of multidisciplinary involvement.^2^

Previous studies showed that goal setting in outpatient CGA was poorly implemented and had poor efficacy for improving well‐being.^14^ Furthermore, an umbrella review of CGA, published in 2018, showed that only one of the included systematic reviews or meta‐analyses of CGA included goal‐setting as a key assessment domain.^2^ In addition, a previous review of goal‐setting in geriatric rehabilitation did not show any evidence of benefit, and the previously mentioned survey of Australian geriatricians showed that only about half (55%) felt goal‐setting to be a core feature of CGA.^7^, ^15^ Interestingly, in the current study, only 47% of letters showed evidence of goals being mentioned, and none of the letters undertook an analysis of long‐versus short‐term goals. However, goal‐setting remains one of the key clinical criteria required by the MBS.^6^ In light of the results of this study and the broader literature, it may be reasonable to reconsider goal‐setting as a requirement for the MBS. This contrasts with the inpatient setting, where goal‐setting is commonly incorporated into CGAs, particularly in the context of multidisciplinary team (MDT) meetings and as it applies to discharge planning.^16^ It is possible that the increased evidence of benefit for CGAs in the inpatient space may be partly explained by goal setting, although further research is required to specifically determine this.^5^, ^17^, ^18^ Future research could investigate this question.

Immunisation history was recorded in less than 50% of the letters in the current study, despite being a core descriptor in the MBS item number. Immunisations are not routinely mentioned in the literature when discussing minimum criteria for CGAs.^2^ Whether it deserves special mention for inclusion in the MBS item number is not clear from the literature. The current study suggests that it is not part of routine clinical practice. In Australia, much of the work of recording and administering vaccinations is done by general practitioners (GPs) and community nurses, rather than specialists. This may explain the relative scarcity of inclusion in the analysed CGAs.

Despite a high number of letters identifying the carer, it was rare for letters to mention carer concerns or carer perspectives. This is in contrast to studies showing that carers like to be involved in planning care and healthcare decision‐making.^19^ Furthermore, it was uncommon in this study for letters to explicitly state what patients had been told. Therefore, it is unclear whether patients are active participants in their CGAs. Person‐centred care is acknowledged as a priority for improvement in healthcare for older adults, although one for which implementation is challenging.^20^ However, these results must be interpreted alongside an acknowledgement that these letters are only a proxy for understanding what happened in the CGAs and will not document all processes. Letters in this form do not act as complete medical documentation but act as communication tools with GPs and medicolegal documents.^21^, ^22^, ^23^

The Australian government has introduced initiatives to increase multidisciplinary working and goal‐focused care. This is in response to acknowledgement of the complex needs of older adults with chronic disease and the importance of integrated care in improving functional outcomes.^24^ Effective teamwork is important in enhancing quality of care and improving functional outcomes.^25^ Multidisciplinary working is thought to be a core feature of CGA.^2^, ^26^ In contrast, the survey of geriatricians found only 60% of geriatricians thought multidisciplinary working was a core feature. In this study, only half of letters included referral to one or more allied health professionals, and none mentioned MDT involvement in care planning or MDT meetings. Notably, multidisciplinary involvement is not stipulated in the MBS minimum criteria for CGA.^6^ This may partly explain the gap between the expectations set in the literature, compared with how CGA is practised by geriatricians. Many of the Australian government initiatives to incentivise multidisciplinary and integrated care focus on GP‐led models rather than specialist or outpatient models of care.^27^ The results of this study provide support for new initiatives and funding models, which support and incentivise multidisciplinary care provided by hospital specialists for older adults.

The themes that emerged in how geriatricians described patient issues were similar to how patients’ concerns were expressed, with a focus on function, future planning, quality of life and symptom management. Previous research showed that patients describe issues as important if they restricted activities of daily living and personal autonomy in social engagement, which is in keeping with the results of this study.^28^

The use of in‐depth quantitative manual content analysis and qualitative thematic analysis is a strength of the study. There are some limitations to note. It is a small sample size of a restricted population. Furthermore, the letters were specific to consultations for CGA for patients with CKD. However, the trial was conducted in all states of Australia, and therefore there is less likely to be bias inferred by restricted geographic areas, although all involved sites were metropolitan. Content analysis of letters is only able to analyse what has been written, not necessarily what has been done. That is, this study could only analyse what was recorded in the letters, not what occurred in the consultation. More complex interactions, such as care planning, are not able to be accurately measured through the methods used in this study. Furthermore, processes might be taking place, such as multidisciplinary working, that the letters do not fully describe. Furthermore, content analysis requires researchers to make somewhat arbitrary definitions; for example, in this study we decided on ‘multidisciplinary involvement’ to mean referral to at least two allied health practitioners. This is higher than the stipulation set by the MBS for team care arrangements, which stipulates the involvement of the GP and at least two other healthcare providers, one of whom can be another medical doctor (meaning referral to only one non‐medical professional is required). This definition is likely broader and more encompassing than the broader literature. Indeed our criteria for ‘multidisciplinary working’ was met any time referrals to multidisciplinary health professionals was made rather than also requiring details on the way those health professionals worked together or the quality of their specific communications.^26^ Another limitation is that no demographic data were available about the geriatricians who wrote the letters, nor was information about the hospitals in which they worked. This impacts the interpretability and generalisability of the results.

## Conclusion

This study provides insight into how CGAs are routinely practised in the outpatient setting, including which clinical domains are routinely incorporated. Despite the letters only serving as a proxy, meaning that processes may have taken place that are not documented in the letters, the results should be considered by geriatricians wanting to improve their practice and health managers looking to incorporate evidence‐based care into outpatient models for older adults. Geriatricians may analyse their practice to consider whether modifications, such as incorporation of goal‐setting or multidisciplinary involvement, might improve outcomes for their patients. There is scope for future research using interviews of geriatricians and patients to understand in depth how this intervention is practised and experienced.

## Appendix I Codebook for descriptive data for manual content analysis of comprehensive geriatric assessment letters in GOAL trial

**Table:**

Variable name	Variable label
collateral	NOK/Carer available (in‐person or telephone) for collateral history. Mark yes if person is available, even if not clear that collateral history has been taken.
Independence	Is patient independent for all ADLs (excluding driving, domestic cleaning and wound management). Gait aid is ok if independent mobility with the aid. Ok if spouse (not child) manages finances as per standard arrangement. Needs to be independent with meal preparation, although ok if in receipt of meal delivery service provided can prepare this independently. Ok if uses a Webster pack.
caregiver	Has a caregiver been identified? Can be formal or informal including family. Mark ‘NA’ if patient is Independent with ADLS as per ‘Independence’
caregiverfamily_concerns	Caregiver or family concerns identified?
caregiver_ability	Assessment of caregiver's ability to care/capabilities (if patient has caregiver; mark NA if no caregiver or caregiver is NA because patient is independent)
caregiver_stress	Assessment of caregiver's stress? (if patient has a caregiver’ mark NA if no caregiver or caregiver is NA because patient is independent)
Standardisedtools	Inclusion of standardised tools. This includes validated screening tools or validated risk stratification tools. Includes scales, questionnaires, etc., but not physical examination (such as routine vital observations) or diagnostic investigations (such as blood tests or radiology). Do not include clock‐face testing unless it is scored numerically. Only include ‘IADL’ if scored numerically. Only include any validated tool if scored numerically.
count_standardisedtools	How many standardised tools were included?
tools	What standardised tools were included? Note that ‘IADL’ counts only if given a numerical/fraction result, not if only used in a descriptive sense.
falls_mobility	Mention of history of falls AND mobility
nutrition	Mention of nutritional status: dietary intake OR appetite, AND weight loss
psychology	Mention of mood or history or mood disorder or psychiatric history
socioeconomic	Mention of (family or social support) AND (education OR occupation history)
medical	Mention of medical history
immunisation	Mention of any vaccinations received OR that immunisations are up to date
medicines	Mention of medicines list
examination	Documentation of clinical examination (more than simply vital observations including postural blood pressure or cognitive testing or frail scale). Do not include if only validated scales such as TUG, grip strength, or gait speed, as these are measured separately in ‘standardisedtools’. Ok if the only mention is ‘sarcopenia’.
pain	Mention of pain
substances	Mention of alcohol AND/OR cigarettes/nicotine. If the only mention is ‘ex‐smoker’ that is ok.
function	Mention of whether the patient is independent with personal activities of daily living (toileting, dressing or bathing) AND instrumental ADLs.
continence	Mention of bowel and/or bladder function
sleep	Mention of sleep
cognition	Mention of cognition or cognitive impairment
capacity	Mention of whether patient has or lacks capacity (or competence) for certain decisions (e.g. health, lifestyle, EPOA). N.B. This relates only to ‘decision‐making capacity’ and not capacity to complete functional tasks/physical limitations. Also, does not relate to ability to do some cognitive tasks, for example, remembering pins/paying bills. It is only about capacity to make decisions
religion	Mention of whether the patient has any spiritual or religious beliefs.
frailty	Is frailty mentioned (not only in describing the GOAL trial)
frailtyscale	What frailty scale is mentioned? If frailty is not mentioned, leave blank. If ‘frail’ is mentioned without a scale, use ‘Arbitrary’
followup	Is there a plan for a follow‐up appointment made or planned for the patient to see the same geriatrician again in clinic. Mark yes only if there is a written plan to follow‐up in the same clinic, mark no if follow‐up is not mentioned or the plan is *not* to follow‐up, or the patient will be followed up by someone else.
medicinechange	Plan for changes to medicines
mdtcollateral	Is collateral or assessments done by allied health professionals available and mentioned?
mdtcareplan	Is there evidence that allied health professionals were involved in making the management plan/care plan?
mdtreferrals	Is there evidence that allied health professionals are referred or delegated to in the management plan/care plan? Includes nursing, for example, older adult mental health referral
multidisciplinary	Role for at least two allied health professionals detailed in the management plan (includes specialist nursing and pharmacy)
mdtreferrals_method	How were referrals facilitated to allied health professionals? Includes specialist teams with a nursing component, for example, older adult mental health services. Do not include memory clinic.
mdtmeeting	Is there mention of an MDT meeting? (at least two other healthcare professionals)
specialists_referrals	Evidence of referrals to other medical specialists? Other than nephrologists.
acp	Discussion of Advanced Health Directive (AHD), statement of choice or advanced care planning more generally, or powers of attorney
patient_concerns	Are the patient concerns identified? If there is a note that the patient has no concerns, mark as ‘no’. If they only specifically mention goals, mark no.
gas	Is there mention of the goal‐attainment scaling that was done as part of the GOAL trial?
goals_general	If GAS was not discussed, were goals discussed more broadly?
goals_shortlong	If goals discussed, broken down into short AND long‐term goals? Need to use these words or similar, not simply state goals that could be short‐ or long term.
patient_told	Is it clear what the patient was told?
behaviours	Were behaviours identified for the patient to change or adopt or reduce? Not simply a task, for example, appointing an enduring power of attorney (EPOA), register with My Aged Care (MAC), etc.
issueslist	Was an issues list included? Can be in paragraph form, for example, an ‘Impression’. Not only past medical history
newdiagnosis	Was a new diagnosis made? This can include geriatric syndromes (including cognitive impairment) or risk fir geriatric syndromes eg falls risk, delirium risk. Can also include new constellation of symptoms that require further workup, for example, concerns around cognition. Can include new issue, for example, low mood due to poor sleep.
managementplan	Inclusion of a management plan included suggestions for patient or GP (or others) for how to address issues.
roles	Inclusion of whose responsibility every point in the management plan is. N.B. An explicit ‘you’ or implied ‘you’ (e.g. Please…) is adequate to mark yes.
headings	Inclusion of (at least three) headings
structured	Issues list plus management plan plus at least three headings
multidimensional	ALL *four* of medical, function(physical), socioeconomic and psychology
Survey_multipledimensions	Medical history *and* Medicine history *and* at least one other domain

## Appendix II Codes phase 2

**Table:**

Codes – Phase 2 generating initial codes
ACP
Active social life
Adjusting to changed health status
Arthritis
Artificial kidney
Balance
Bone health
Caring responsibilities
Chronic health conditions
Cognitive Impairment
Comorbidities
Constipation
Continence
Crafts
Declining function
Dialysis
Difficulty concentrating
Dysphagia
Dyspnoea
Dyspnoea or fatigue
Oedema
Education and knowledge
Falls
Fatigue
Fitness
Fitness for surgery
Frailty
Function
Functional decline
Gardening
General worry about health
General worry about the future
Healthy lifestyle
Home modification
Kidney transplant
Leisure and enjoyable activities
Loneliness
Longevity
Maintaining independence
Medical investigations
Medical risk factors
Medication management
Mobility
Mood
Multidisciplinary
New medical diagnoses
Nutrition
Obesity
Pain
Polypharmacy
Preventing deterioration
Pruritus
Quality of life
Reduced strength
Sarcopenia
Sensory impairment
Sleep
Social life
Social support
Stay independent
Support for ADLs
Surgery or medical treatment
Sweat
Time and finance impact of healthcare
Too many medical appointments
Travel
Vision changes
Workforce participations
Writing

## Appendix III Codes phase 3

**Table:**

Codes – Phase 3 Searching for Themes
Geriatrician issues list
Advanced care planning
Chronic health problems
Diagnosis of new medical issues
Dysphagia
Hearing vision impairment
Investigations
Osteoporosis
Sensory neuropathy
Investigations
Listing of previously known medical issues
Manage comorbidities
Management of medical risk factors
Medicine management
Prevention of disease states
Cognition
Falls and mobility
Build strength
Falls
Fitness
Mobility
Frailty
Healthy lifestyle
Nutrition
Independence and quality of life
Management of functional impairment
Low mood psychological adjustment to changed health status
Obesity
Social
Hobbies and meaningful activities
Loneliness
Social engagement
Support for ADLs
Environmental modification to support independence or function
Support for activities of daily living
Symptom identification and management
Breathlessness
Dysphagia
Oedema
Fatigue
Pain management
Physical symptoms
Poor sleep quality or quantity
Pruritus
Patient concerns
Advanced care planning
Arthritis
Cognition or memory concerns
Concerns around how will meet caring responsibilities in future
Constipation
Continence
Decline in function
Dialysis‐related
Negative impact on well‐being
Nutrition
Time and budget
Diet or nutrition
Difficulty concentrating and difficulty with attention
Dysphagia
Dyspnoea or fatigue
Falls or falls risk
Fatigue
Fatigue (related to dialysis)
Financial concerns
Financial impact of healthcare
Fitness
Fitness for surgery
Frailty
General concerns around health
Grief or loneliness
Impact of chronic health conditions on quality of life
Leg cramps
Low mood
Low mood due to adjustment to reduced abilities or disease state
Low mood due to health status
Medicines side effects
Mobility
New code
New code (2)
New code (3)
Social isolation
Pain
Participation in hobbies
Physical fitness
Physical slowing
Pruritus
Quality of life
Reduced quality of life related to health problems
Sleep quality or quantity
Stress related to carer roles
Weight
Patient goals
Access to equipment
Achieve surgery for knee replacement
Active workforce participation
Artificial kidney or kidney transplant
Be in charge of managing health
Cognition improvement or maintenance
Community service
Complete advanced care planning
Diet or nutrition
Education
Financial
Fitness for surgery
Improve balance
Improve fitness
Improve mobility
Improve pain
Improve quality of life
Increase activity or hobby
Increase exercise
Increase knowledge about healthcare
Increase support
Longevity
Maintain a good lifestyle
Maintain independence
Maintain social engagement
Mood
Patient goals
Access to equipment
Achieve surgery for knee replacement
Active workforce participation
Artificial kidney or kidney transplant
Be in charge of managing health
Cognition improvement or maintenance
Community service
Complete advanced care planning
Diet or nutrition
Education
Financial
Fitness for surgery
Improve balance
Improve fitness
Improve mobility
Improve pain
Improve quality of life
Increase activity or hobby
Increase exercise
Increase knowledge about healthcare
Increase support
Longevity
Maintain a good lifestyle
Maintain independence
Maintain social engagement
Mood
Reduce alcohol intake
Reduce polypharmacy
Slow progression of chronic disease
Social engagement
Social isolation
Travel
Workforce participation
Reduce alcohol intake
Reduce polypharmacy
Slow progression of chronic disease
Social engagement
Social isolation
Travel
Workforce participation

## Appendix IV Codes phase 4

**Table:**

Codes – Phase 4 Reviewing Themes
Geriatrician issues list
Advanced care planning
Cognitive impairment diagnosis and strategies to maintain cognitive health
Lifestyle modification and optimisation
Healthy lifestyle
Obesity
Low mood
Managing medical problems
Chronic health problems
Manage comorbidities
Management of medical risk factors
Medicine management
New medical issues
Dysphagia
Hearing vision impairment
Investigations
Osteoporosis
Strategies to manage frailty and functional decline
Falls and mobility
Build strength
Falls
Mobility
Fitness
Frailty and functional decline
Frailty
Functional decline
Nutrition
Sarcopenia
Strategies to support independence, quality of life and psychosocial well‐being
Low mood psychological adjustment to changed health status
Management of functional impairment
Psychosocial strategies to improve quality of life
Hobbies and meaningful activities
Social engagement
Strategies to address loneliness
Strategies to support independence or function
Environmental modification to support independence or function
Support for activities of daily living
Symptom identification and management
Breathlessness
Dysphagia
Oedema
Fatigue
Pain management
Physical symptoms
Poor sleep quality or quantity
Pruritus
Patient concerns
Advanced care planning
Cognition or Memory concerns
Difficulty concentrating and difficulty with attention
Fatigue
Fatigue (related to dialysis)
Frailty
Physical slowing
Function
Continence
Decline in function
Falls or falls risk
Mobility
Physical fitness
General concerns around health
Dialysis‐related
Negative impact on well‐being
Nutrition
Time and budget
Healthy lifestyle
Diet or nutrition
Fitness
Fitness for surgery
Weight
Impact of chronic health conditions on quality of life
Medicines side effects
Low mood
Grief or loneliness
Low mood due to adjustment to reduced abilities or disease state
Low mood due to health status
Quality of life
Participation in hobbies
Reduced quality of life related to health problems
Social isolation
Symptoms
Arthritis
Constipation
Dysphagia
Dyspnoea or fatigue
Leg cramps
Pain
Pruritus
Sleep quality or quantity
Social
Carer responsibilities
Concerns around how will meet caring responsibilities in future
Stress related to carer roles
Financial concerns
Financial impact of healthcare
Patient goals
Cognition improvement or maintenance
Complete advanced care planning
Education
Increase knowledge about healthcare
Financial
Healthcare attainment
Achieve surgery for knee replacement
Artificial kidney or kidney transplant
Fitness for surgery
Longevity
Reduce polypharmacy
Slow progression of chronic disease
Healthy lifestyle
Diet or nutrition
Increase exercise
Maintain a good lifestyle
Improve quality of life
Improve pain
Meaningful activities
Active workforce participation
Community service
Increase activity or hobby
Maintain social engagement
Travel
Workforce participation
Social engagement
Social isolation
Increase support
Access to equipment
Independence
Be in charge of managing health
Improve mobility
Improve balance
Improve fitness
Maintain independence
Mood

## Appendix V Codes phase 5

**Table:**

Codes – Phase 5 defining and naming themes	
Geriatrician issues list	
Advanced care planning	Issues related to planning future care
Planning for future care requirements	Issues relating to planning for future care requirements for self or family
Planning for future medical care	Issues related to planning future medical care
Substitute decision‐makers	Issues relating to identifying and appointing substitute decision‐makers
Identification and management of medical comorbidities and risk factors	Issues relating to the diagnosis or identification of medical issues including comorbid health conditions, and risk factors for disease states
Cognitive impairment diagnosis and strategies to maintain cognitive health	Issues relating to the diagnosis or concerns relating to cognition or cognitive decline, and risk for cognitive decline
Identification and management of non‐cognitive comorbidities	Issues relating to the diagnosis and management of medical issues that are not cognitive in nature
Strategies to manage frailty and functional decline	Issues relating to managing the progression and effects of frailty and declining function
Frailty and functional decline	Issues relating to managing frailty and functional decline
Mobility and falls	Issues relating to managing change in mobility, falls and falls risk
Strategies to support independence, quality of life and psychosocial well‐being	Issues relating to the need to support or maintain independence, quality of life and psychosocial well‐being
Low mood including psychological adjustment to changed health status	Issues relating to low mood
Psychosocial strategies to improve quality of life	Issues relating to psychological or social strategies that could improve quality of life
Strategies to support independence or functioning	Issues relating to identifying strategies that support independence and function
Symptom identification and management	Issues relating to the identification and management of symptoms
Fatigue and breathlessness	Issues relating to the identification and management of fatigue and breathlessness
Non‐fatigue symptoms	Issues relating to the identification and management of non‐fatigue symptoms
Patient concerns	
Cognition and Mood Concerns	Patient concerns relating to mood and cognition
Cognition or Memory concerns	Patient concerns relating to cognition or memory
Mood Concerns	Patient concerns relating to low mood
Declining function and difficulties with managing activities of daily living	Patient concerns relating to declining function and difficulties managing activities of daily living
Falls	Patient concerns relating to falls
Physical slowing and functional decline	Patient concerns relating to physical slowing and functional decline
Future Planning	Patient concerns relating to planning for the future
Barriers to accessing care and support	Patient concerns relating to barriers to accessing care and/or support
Concern around how will meet responsibilities in future	Patient concerns about how will meet future responsibilities
Impact of medical care on quality of life	Patient concerns about the impact of medical care on quality of life
*Not included* Education about lifestyle modifications to optimise health	
Diet and Nutrition	
Symptom management	Patient concerns around symptom management
Fatigue and dyspnoea	Patient concerns about management of fatigue and dyspnoea
Non‐fatigue symptoms	Patient concerns around management of non‐fatigue symptoms
Patient goals	
Improve health status	Patient goals about improving health status
Lifestyle modification to improve health or fitness	Patient goals about lifestyle modification to improve health and/or fitness
Medical treatment	Patient goals about accessing or completing medical treatments including surgery
Increase or maintain engagement	Patient goals about increasing or maintaining engagement in activities
Social engagement	Patient goals about increasing or maintaining social engagement
Work or leisure activity	Patient goals about increasing or maintaining work or leisure activities
Maintain independence and personal agency	Patient goals about maintaining independence or control over one's life
Increase support	Patient goals around accessing increased support to maintain independence
Live independently	Patient goals around continuing to live independently

## Appendix VI Narrative summary of qualitative analysis (phase 6 of reflexive thematic analysis)

### Geriatrician issues

Identification and management of medical comorbidities was an identified theme. This most occurred in the form of cognitive assessments and consideration of a diagnosis of cognitive impairment or dementia. These cognitive assessments were a key feature of the letters, and in letters an impression of cognitive impairment could be made in people who had no prior history of cognitive impairment. Mood disorders and osteoporosis were other diagnoses commonly made or considered.

#### Illustrative quotes

[Impression:] multi‐domain cognitive impairment … MoCA test today 21/30 with recall difficulties.

He does have some subjective short‐term memory impairment, though there are reassuring features including a MMSE score of 29/30 today, lack of progression over >5 years and a lack of functional impairment due to these concerns. I do wonder if his short‐term memory concerns are primarily related to his anxiety (with associated inattention).

Please arrange a bone mineral density scan looking for osteoporosis given his risk of renal osteodystrophy and osteoporosis.

Alongside this focus on health conditions, there was a focus on symptoms, and symptom burden, experienced by the patients. Fatigue, pain and dyspnoea were frequently mentioned in the context of causing significant symptom burden.

#### Illustrative quotes

[Patient has] progressive fatigue noted on high level activity, particularly on lawn mowing and yard keeping.

In regard to his analgesia and ongoing lower limb neuropathic pain of 6/10, which is probably due to his peripheral neuropathy, we did discuss the ongoing use of pregabalin. He is currently on pregabalin 25 mg twice daily. Paracetamol does not seem to provide much relief. I wonder if we should increase his evening dose pregabalin to 50 mg while maintaining the same morning dose.

Advance care planning was another issue mentioned in the letters. This was often presented in the body of the letter with regards to what advanced care planning decisions had been done previously, but was also mentioned in the impression, often recommending that the patient or GP undertake advanced care planning. It was often mentioned with regards to appointment of an enduring power or attorney or guardianship, but also included mention of previous decisions that had been made about limiting future medical treatment and priorities for future care. Occasionally letters mentioned a discussion that the geriatrician had directly undertaken with the patient about their wishes with regards to healthcare and future treatments.

#### Illustrative quotes

Please continue to encourage [patient] to complete advanced care planning by completing an EPOA and AHD.

During her admission with COVID, she did assess her goals of care … At present, she is documented as ‘for resuscitation’. On conversation today, she was undecided as to whether she would want CPR if her heart actually stopped. We agreed that it's better if she makes these decisions in advance…

There was a particular focus in the letters on strategies to identify and manage frailty and functional decline. At times this focused specifically on frailty, with recommendations to focus on resistance training and nutrition. At other times the focus was more on falls or falls risk, with recommendations to improve or maintain mobility through balance training, physiotherapy or other regular exercise.

#### Illustrative quotes

[H]high risk of falls in the setting of modifiable risk factors of untreated chronic pain, fatigue, polypharmacy, and environmental factors (encouraging use of a walking stick and appropriate footwear). Non‐modifiable risk factors include osteoarthritis and peripheral neuropathy.

She is a significant falls risk which is likely to be multifactorial due to her poor balance, neuropathy, central aetiology as well as polypharmacy. She would benefit from a structured exercise program; I will refer her onto the [Hospital Name] Health Campus’ [Falls Prevention] program.

My impression is that [Patient] has multimorbidity and is frail.

Following on from this focus on function, the letters often highlighted the importance of maximising independence and quality of life. This included discussion about low mood or depression, and the impact of changing health status on mood and quality of life. Letters focused on social engagement and meaningful activities as avenues of supporting social well‐being and quality of life.

#### Illustrative quotes

Encouraged [patient] to pursue his in interests – previously avid singer and guitar player but gave it up due to loss of dexterity/grip in his fingers.

[Patient] would likely benefit from increased contact with family, and she will think about how this can be safely and practically achieved.

### Patient concerns

Letters mentioned patient concerns around declining memory and cognition. This was often mentioned alongside, or in addition to, concerns around low mood and psychological well‐being. Furthermore, letters often described patients’ anxiety or low mood as a result of perceived cognitive changes.

#### Illustrative quotes

[H]e reports long‐standing short‐term memory complaints, particularly with respect to being repetitive in asking questions as well as losing items around the house.

Cognitively, [Patient] feels that his short‐term memory has deteriorated over the course of the last year. In particular, he finds it easy to misplace items around the house, particularly tools when he is working on something. He finds this frustrating as he spends much time trying to relocate things. He is otherwise not concerned about his working memory.

This theme tied in with another, of patient concerns about declining function and increased dependence. Letters mentioned not only falls, but physical slowing, as patient concerns.

#### Illustrative quotes

[H]e has become more irritable in the past few years, primarily because he is not able to do the things he wants to (such as gardening) due to low energy and pain.

Mood: Pt reported. is feeling fatigued all the time – sleeping well at night but constantly tired. [Patient] doesn't have capacity to manage more things and feels its ‘all been a bit much’ with one thing after the next.

This led onto the next themes which was future planning. Patients were described to have concerns around accessing care and support, and concerns about how they will meet future caring responsibilities, in particular planning for future care of a spouse or dependent. They also had concerns about the impact of medical care on quality of life. In particular, patients mentioned concerns around the time or financial costs of dialysis.

#### Illustrative quotes

Worries about health and kidney – HD…wonders how going to fit in with budget and time management.

My biggest concerns, if something happened to me, I am worried about… our son [Who has significant health issues]… Worries about her future and health a lot.

[Patient] worries that he would like his wife to be supported in the event that he has a deterioration in his health.

Finally, similarly to the geriatricians’ issues, patients’ concerns were often mentioned related to symptoms. Ther was often a focus on pain, which was perceived as being separate to the CKD, or fatigue, which was often mentioned in the context of CKD or dialysis. Other symptoms included pruritus, constipation and breathlessness.

#### Illustrative quotes

He complains of lower limb neuropathic pain from 6–8/10 and hence he is on a small dose of pregabalin.

…low mood (see below), and chronic bad pain in neck and shoulders, which impair her WQOL, in addition to more minor niggles from arthritis in her other joints, for example, knees.

### Patient goals

Patient goals were often represented as improving health status. This was in the form of lifestyle modification, such as increasing regular physical activity, improving nutrition or reducing alcohol. It also related to medical treatment, for example, goals around attaining surgery for arthritis or a renal transplant.

#### Illustrative quotes

…was also seen by [Doctor] today and she reports having a goal of participating in an artificial kidney trial.

[Patient] is compliant with his therapy and hoping to receive a kidney transplant in the future.

Another way in which patient goals were represented was increasing engagement. This included social engagement, such as seeing family more regular or maintaining an active social life. It also focused on leisure or work, such as re‐entering the workforce or increasing time doing a hobby such as gardening, writing or crafts.

#### Illustrative quotes

Feeling more energetic since commencing dialysis. Planning to do some small trips. Previously travel[l]ed extensively…

He would like to remain as independent as possible and still actively participates in his business.

[Goal is] to maintain her social outings with weekly lunch with family and friends. She loves social group activities.

The other theme that came through for patient goals was maintaining independence. This often was discussed in terms of functional independence and remaining living at home, including obtaining the necessary support needed to do so. This theme also extended to maintenance of personal autonomy and being ‘in control’ of the decisions and directions in one's life.

#### Illustrative quotes

…does not have any specific concerns about his cognition but is wanting to remain as cognitively sharp as possible for as long as possible.

I would like to know what is going on and plan my life around, Like to be in control and to take less medications if possible.

…main goals at present are to continue to remain in her own home in the community, and for her renal function to remain stable, so that she can continue her hobby creating soft toys and quilts…
